# Cyanobacterial blooms at Canadian freshwater beaches: A prospective cohort study of exposure, health outcomes, and One Health implications

**DOI:** 10.1016/j.onehlt.2026.101608

**Published:** 2026-10-09

**Authors:** Binyam N. Desta, Jordan Tustin, Rachel Jardine, Claire Holeton, Teresa Brooks, Elizabeth Montgomery, Dylan Lyng, J. Johanna Sanchez, Fatih Sekercioglu, Ian Young

**Affiliations:** a Department of Population Medicine, University of Guelph, Guelph, Ontario, Canada; b School of Occupational and Public Health, Toronto Metropolitan University, Toronto, Ontario, Canada; c Environmental Monitoring and Reporting Branch, Ontario Ministry of the Environment, Toronto, Ontario, Canada; d Water and Air Quality Bureau, Health Canada, Ottawa, Ontario, Canada; e Halifax Regional Municipality, Halifax, NS, Canada; f Environment and Climate Change, Government of Manitoba, Winnipeg, Manitoba, Canada

**Keywords:** Cyanobacteria, Blue-green algae, Harmful algal bloom, Characteristics, Recreational water, Illness, exposure, *E.coli*

## Abstract

Cyanobacterial blooms are an emerging One Health concern in freshwater environments, yet limited evidence exists on how humans and companion animals interact with bloom-prone waters or how these exposures align with water-quality indicators. This study characterizes recreational behaviours, environmental conditions, and self-reported illness patterns across multiple Canadian beaches to inform integrated monitoring and risk-management approaches. A prospective cohort study was conducted at seven freshwater beaches in Manitoba, Nova Scotia, and Ontario during the 2024 and 2025 summer seasons. Participants completed on-site surveys detailing demographics and water-contact behaviours, followed up 3-7 days later with an illness survey. Dog exposures and illnesses were also recorded. Cyanobacterial cell counts, pigments, and *E. coli* counts were measured on recruitment days. Descriptive analyses and exploratory visualizations examined patterns across human, dog, and environmental data. Among 2,324 participants, water exposure patterns varied widely across sites and demographic groups, with children and teenagers demonstrating the highest water-contact behaviours. Dogs exhibited similar high-contact patterns. Cyanobacterial concentrations showed substantial spatial and temporal variability and did not correlate with *E. coli* counts. Approximately two-thirds of participants (66.6%) completed the illness follow-up survey. Exploratory analyses revealed a basic association between cyanobacteria and acute gastrointestinal illness (AGI), although the incidence of AGI was low in both humans and dogs. Freshwater recreational environments represent shared human–animal–microbial interfaces. Multi-indicator monitoring, including cyanobacterial metrics, and targeted communication for high-contact groups are essential. A One Health approach integrating human, veterinary, and environmental surveillance will be critical as cyanobacterial blooms intensify under changing climatic conditions.

## Highlights

• Recreational exposures varied widely across age groups and sites.
• Children, teenagers, and dogs had the highest water and sand contact.
• Cyanobacteria levels varied and were not correlated with *E. coli* counts.
• Multi-indicator, One Health monitoring is needed for cyanobacterial bloom risks.

## 1 Background

Harmful algal blooms (HABs), particularly cyanobacterial blooms, pose a growing One Health concern worldwide in freshwater ecosystems [1], [2]. Cyanobacteria can proliferate rapidly under warm, nutrient-rich conditions and produce cyanotoxins that can affect humans, animals, and wildlife [2], [3], [4], [5], [6]. In Canada, bloom occurrence has increased in recent decades, with reports concentrated during summer months when recreational water use peaks [7], [8]. Concurrently, changing climate patterns, including rising temperatures and more frequent extreme precipitation, are projected to intensify bloom dynamics and expand their geographic distribution [9], [10], [11].

Human exposure to cyanobacteria may occur through skin contact, accidental ingestion, or inhalation of aerosolized particles, leading to a wide spectrum of health effects ranging from mild irritation and gastrointestinal symptoms to severe hepatic and neurologic toxicity [4], [12], [13], [14], [15]. Domestic animals, particularly dogs, are highly exposed because they play/drink in waters [15], [16].

Despite recurring swimming advisories (based on Canadian guidelines for recreational water quality addressing cyanobacteria and their toxins along with fecal indicator bacteria such as *E. coli* counts [17]), limited empirical evidence exists on how Canadians interact with recreational water during bloom-prone periods, the extent of resulting exposure, and the incidence of associated health outcomes among humans and dogs

Despite ongoing swimming advisories based on Canadian recreational water quality guidelines, there is limited empirical evidence on how Canadians engage with recreational water during periods prone to cyanobacteria blooms [[7], [17], [18]]. More specifically, there is insufficient information on the extent of exposure and the incidence of related health issues in both humans and dogs [[7], [18]]. For example, a 2009 study is the only published research in Canada to examine the health effects of cyanobacteria on residents living near recreational areas. The study area included three lakes in Quebec and found that exposure to cyanobacterial cells through various recreational activities, including limited secondary-contact activities such as boating and fishing, increased the risk of gastrointestinal illness [19]. Importantly, recreational exposure is not limited to swimming or intentional ingestion of water. Activities involving immersion or submersion, water sprays and aerosols, and water-based sports can create opportunities for exposure through ingestion, inhalation, and direct contact with the skin, eyes, and other mucous membranes [[12], [20], [21]]. Data on companion animal health was not collected.

This prospective cohort study integrates environmental monitoring with human and animal exposure and health outcome data to address these gaps and inform One Health-aligned public health strategies. This study also provides evidence to inform targeted intervention strategies, enhance One Health surveillance, and guide policies to reduce the burden of cyanobacteria-related illness in Canada.

## 2 Methods

The study design and planned methods were previously described in a protocol published before data collection[22]. The present study reports the empirical findings from implementing that protocol. Below is a summary of the methods relevant to the current analyses.

### 2.1 Study Design and Setting

A multi-site, prospective cohort study was conducted during the summer months of 2024 and 2025 at seven freshwater recreational beaches across three provinces in Canada: Ontario, Manitoba, and Nova Scotia. The selected beaches include (grouped into four sites): (1) Grand Beach West and East (Manitoba), (2) Shubenacadie Canal, Shubenacadie Dog Beach, and Kinsmen (Halifax, Nova Scotia), (3) Kinsmen in Port Perry (Ontario), and (4) Colchester in Windsor-Essex (Ontario). The beaches in Manitoba and Halifax were grouped together due to their proximity, while the beaches in Ontario were not grouped because they are located in distinct areas. The Kinsmen beach in Halifax was added mid-season in 2025 due to closure of the dog beach site as a result of wildfire activity. The sites and beaches were identified based on their history of cyanobacterial blooms, anticipated bloom risk, and popularity for recreational water use. This geographic diversity enabled characterization of a range of exposure scenarios and environmental conditions relevant to Canadian freshwater systems. The selected beaches represent a range of freshwater coastal and inland recreational environments across Canada. Grand Beach West and East in Manitoba are located within Grand Beach Provincial Park, along Lake Winnipeg, and are characterized by extensive white-sand shorelines, shallow waters, and dune systems that support high recreational use [23]. In Nova Scotia, the Shubenacadie Canal, Shubenacadie Dog Beach, and Kinsmen sites are situated within the Shubenacadie Canal corridor, a historic waterway linking Halifax Harbour to the Bay of Fundy, and are primarily freshwater recreational areas used for swimming and paddling [24]. Kinsmen Beach in Port Perry lies on Lake Scugog and functions as a small municipal beach with designated recreational use other than swimming [25]. Finally, Colchester Beach in Windsor-Essex, located along Lake Erie, features a sandy shoreline and adjacent parkland, making it a popular regional destination for swimming and lakeside activities [26].

### 2.2 Participant Eligibility and Enrollment

Recreational water users of any age were eligible to participate if they could provide informed consent, complete the study surveys in English or French, and had not participated in the study within the preceding 21 days. Accompanying dogs were also eligible for inclusion through their participating owners. In line with the acute and self-limiting nature of the outcomes being studied, a 21-day washout period was allowed for re-enrollment. Recruitment occurred over 24 days per site, approximately twice weekly, over a 12-week period across two summer seasons.

Trained data collectors approached individuals at the main beach access points throughout each recruitment day. Households with children and/or dogs were prioritized for recruitment. Informed consent was obtained from all adult participants, with parental consent and child assent secured for minors. Each participating household received a CAD $10 gift card as an incentive.

### 2.3 Data Collection

Data collection consisted of on-site surveys and follow-up survey. On-site survey data were collected using tablet-based structured questionnaires administered through SimpleSurvey (Quebec, Canada). Trained data collectors approached recreational water users throughout the day, obtained informed consent, and administered a structured ‘Beach Survey’ via tablet at the end of the recreational water users’ beach visit. This survey captured sociodemographic characteristics, recreational activities, exposure to water and sand, beach visit duration, and any baseline relevant health symptoms.

Participants were recontacted 3 – 7 days later to complete the ‘Follow-Up Survey,’ which assessed new-onset symptoms within 3 days of their beach visit, consistent with previous symptom onset windows following exposure to cyanobacterial toxins [2]. Participants were contacted using their preferred communication method, i.e., email, SMS, or phone call, to complete the follow-up survey.

Based on the follow-up survey, the primary health outcomes self-reported by human participants included gastrointestinal and respiratory illnesses, skin and eye infections, and generalized symptoms such as fatigue and headache. For dogs, the assessed outcomes included gastrointestinal symptoms (vomiting and diarrhea), dermatologic symptoms (rash and itching), neurologic symptoms (seizures and lethargy), and general illness (such as weakness and appetite loss).

### 2.4 Water sample collection and analysis

Data collection also included environmental sampling on each recruitment day, where exposure to cyanobacteria was assessed through direct water sampling, with two water samples collected per beach daily for microscopic analysis of cyanobacterial cell counts (cells/mL). The samples were delivered to the local laboratory in each province for immediate analysis, and in Windsor-Essex, they were preserved in Lugol’s iodine and shipped in batches, a standard procedure to fix cells and preserve detectability during storage and shipment to the York Durham Regional Environmental Laboratory. In the field, rapid test strips (ABRAXIS Rapid Immunochromatographic Test, Gold Standard Diagnostics, Horsham, US) were employed to screen for the presence or absence of total microcystins. Additionally, as part of the screening process, data collectors identified visible cyanobacterial blooms based on surface accumulations or water discoloration consistent with cyanobacterial proliferation. Because cyanobacterial blooms may also occur as dispersed populations without visible surface accumulations [27], these observations were limited to visible bloom events and do not indicate the absence of non-visible blooms. Confirmatory quantification was then carried out using ELISA methods. Handheld fluorometers measured concentrations of chlorophyll a and phycocyanin, providing insight into bloom-related pigments. Due to technical difficulties with the fluorometer devices at the Manitoba and Halifax sites, we report only data from the two Ontario sites. Water samples were collected and analyzed for generic *E. coli* counts using standard culture methods. Participants’ recreational water contact was categorized as no contact (control), minimal contact (e.g., wading or boating), or full-body immersion (e.g., swimming).

Environmental sampling and laboratory analyses characterized the environmental conditions and cyanobacterial bloom events encountered during the study period. For the present study, these measurements were descriptive and were not used to assess associations between microcystin concentrations and self-reported health outcomes.

### 2.5 Data Analysis

Descriptive statistics were generated to characterize participants, dogs, water-contact behaviours, and self-reported illness outcomes across study sites. Categorical variables were summarized as frequencies and percentages, and continuous variables as means with standard deviations or medians with interquartile ranges, as appropriate. For descriptive purposes, participants were grouped into water-contact and no-contact, and into those who swallowed water and those who did not, and cross-tabulated with acute gastrointestinal illness (AGI). Cyanobacterial cell counts were summarized across sites and sampling dates. A phycocyanin-to-chlorophyll-a ratio variable was created as a supplementary indicator of the relative contribution of cyanobacterial to phytoplankton biomass, recognizing that phycocyanin is not exclusive to cyanobacteria [[28], [29], [30]]. Pearson correlation coefficients were used to assess relationships between cyanobacteria, *E. coli* counts, and pigment (phycocyanin-to-chlorophyll a) ratios. All analyses were conducted using RStudio (R version 4.4.2). This study’s formatting and analysis of R script files are available from the following GitHub page (the dataset will be made available after the study team completes all planned analyses): https://github.com/bndesta/algal_bloom

### 2.6 Ethics

This study received ethical approval from the Toronto Metropolitan University Research Ethics Board (Protocol #2023-461). All participants provided informed consent prior to enrolment, with parental consent and child assent obtained for minors. For participants under 18, consent procedures were conducted with age-appropriate communication and comprehension. Participants were informed of the voluntary nature of the study, their right to withdraw at any time, and the steps taken to ensure data confidentiality and secure storage. Contact information for the research team and the ethics board was provided in case of questions or concerns.

## 3 Results

### 3.1 Participant characteristics

Across the four study sites, 2,324 recreational water users were enrolled during the 2024 and 2025 summer seasons, with the largest proportion recruited in the two Ontario sites (47%) and Manitoba (41%) (Table 1). The follow-up survey completion rate was 66.6%. Participant demographics varied across the four study sites, with notable differences in the representation of children and adolescents (Table 1). Children aged 0–14 years comprised roughly one-fifth of participants at the Manitoba (21%) and Halifax (17%) sites, but a much smaller share at Port Perry (10%). Teen participation (15–19 years) showed similar variation, ranging from 1% in Halifax to 17% in Windsor-Essex. Gender distributions were largely consistent across sites, with women and girls comprising 58 – 60% of participants at most locations, while Port Perry exhibited an almost equal gender split. Educational attainment differed markedly, with Halifax having the highest proportion of households holding postgraduate degrees (44%). Ethno-racial diversity was most significant at Port Perry, where East Asian (25%) and South Asian (14%) participants were more strongly represented than at other sites. Baseline illness was infrequent overall, with respiratory conditions (5 – 9%) and allergies (8 – 11%) reported most commonly across sites (Table 1).

**Table 1:** Recreational water users’ characteristics across three provinces of Canada, stratified by site, 2024-2025

Characteristic	Manitoba (2024) N (%) (N = 958)	Halifax (2025) N (%) (N = 278)	Port Perry – ON (2024) N (%) (N = 523)	Windsor-Essex – ON (2025) N (%) (N = 565)	Total N (%) (N = 2324)
Age group of participants (years)					
0-14	196 (20.5)	48 (17.3)	52 (10.1)	79 (14.0)	375 (16.2)
15-19	95 (9.9)	4 (1.4)	32 (6.2)	95 (16.8)	226 (9.8)
20-39	231 (24.1)	78 (28.1)	160 (31.0)	215 (38.1)	684 (29.5)
40+	435 (45.5)	148 (53.2)	272 (52.7)	176 (31.2)	1,031 (44.5)
Prefer not to respond	1	0	7	0	8
Gender identity					
Woman/girl	566 (59.9)	161 (58.3)	255 (49.6)	317 (57.0)	1,299 (56.7)
Man/boy	373 (39.5)	111 (40.2)	254 (49.4)	237 (42.6)	975 (42.6)
Gender fluid, non-binary, or transgender	6 (0.6)	4 (1.4)	5 (1.0)	2 (0.4)	17 (0.7)
Prefer not to respond	13	2	9	9	33
Highest education completed in household					
High school or less	157 (16.9)	29 (10.5)	70 (14.0)	95 (17.4)	351 (15.6)
College, trades, or apprenticeship	201 (21.6)	50 (18.1)	114 (22.8)	170 (31.2)	535 (23.8)
Bachelor’s degree	267 (28.7)	76 (27.4)	174 (34.9)	113 (20.7)	630 (28.0)
Post-graduate degree	304 (32.7)	122 (44.0)	141 (28.3)	167 (30.6)	734 (32.6)
Prefer not to respond	29	1	24	20	74
Ethno-racial identity					
White	718 (78.0)	239 (88.8)	229 (45.3)	418 (77.3)	1,604 (71.7)
South Asian	10 (1.1)	4 (1.5)	70 (13.8)	11 (2.0)	95 (4.2)
East Asian	7 (0.8)	4 (1.5)	126 (24.9)	10 (1.8)	147 (6.6)
Arab	1 (0.1)	0 (0.0)	33 (6.5)	31 (5.7)	65 (2.9)
Southeast Asian	20 (2.2)	1 (0.4)	16 (3.2)	3 (0.6)	40 (1.8)
Latin	14 (1.5)	2 (0.7)	9 (1.8)	23 (4.3)	48 (2.1)
Black	13 (1.4)	8 (3.0)	4 (0.8)	15 (2.8)	40 (1.8)
Multiple ethnicities	131 (14.2)	11 (4.1)	18 (3.6)	30 (5.5)	190 (8.5)
Indigenous	6 (0.7)	0 (0.0)	1 (0.2)	0 (0.0)	7 (0.3)
Prefer not to respond	38	9	17	24	88
Baseline illness status^1^					
Respiratory illness	82 (8.6)	18 (6.5)	32 (6.1)	30 (5.3)	162 (7.0)
AGI	31 (3.2)	4 (1.4)	5 (1.0)	11 (1.9)	51 (2.2)
Skin infection	17 (1.8)	6 (2.2)	4 (0.8)	6 (1.1)	33 (1.4)
Eye infection	10 (1.0)	1 (0.4)	0 (0.0)	7 (1.2)	18 (0.8)
Generalized illness	79 (8.2)	27 (9.7)	35 (6.7)	43 (7.6)	184 (7.9)
Baseline health conditions^1^					
Allergies	87 (9.1)	30 (10.8)	47 (9.0)	45 (8.0)	209 (9.0)
Chronic skin condition	74 (7.7)	15 (5.4)	14 (2.7)	20 (3.5)	123 (5.3)
Chronic respiratory condition	49 (5.1)	14 (5.0)	18 (3.4)	22 (3.9)	103 (4.4)
Chronic gastrointestinal condition	35 (3.7)	7 (2.5)	13 (2.5)	9 (1.6)	64 (2.8)
Immuno-compromised	14 (1.5)	12 (4.3)	8 (1.5)	11 (1.9)	45 (1.9)

1 Multiple selections were possible for these variables

### 3.2 Water exposure

Recreational exposure patterns varied across the four study sites. Manitoba showed the highest water-contact activity, with 69% entering the water and 55% swimming, compared with very low participation in Halifax (15%) and Port Perry (8%). Windsor displayed intermediate but substantial activity, particularly for swimming (41%) and head-submersion (20%). Sand contact was common in Manitoba (50%) and Windsor (47%), but rare in Halifax (18%) and especially Port Perry (4%). Notably, Halifax had lower algae or seaweed contact (5%) than higher levels (26–33%) at other sites (see Table S1).

Across all beaches, children demonstrated the highest rates of exposure for water and sand contact (Figure 1). Among participants aged 0–14, more than 55% engaged in swimming and over 70% in sand contact, compared with considerably lower frequencies among adults ≥40 years. Overall water-contact rates were similar between genders, although men and boys more frequently reported activities involving head submersion (Figure 2). Exposure patterns also varied across sociodemographic groups. Participants from households with a high-school education or less demonstrated the highest overall rates of any water contact (51%), swimming (42%), and head-underwater activities (25%), whereas those with higher educational attainment showed lower swimming prevalence (32 – 34%) and only slightly higher rates of wading (Figure 3). Differences across ethno-racial groups were substantial. White participants had the highest prevalence of swimming (35%) and full-body immersion (20%) among the large population groups, while South Asian and East Asian participants reported markedly lower swimming (15% and 4%, respectively) and head-underwater exposure (8% and 2%). Several smaller groups exhibited distinct patterns: Latin (46%), Black (50%), and participants reporting multiple ethnicities (55%) showed relatively high swimming rates, and Indigenous participants showed some of the highest rates of full immersion (43%), though numbers were small (Figure 4). These patterns highlight considerable heterogeneity in recreational water behaviour across sociodemographic categories.

**Figure 1:**
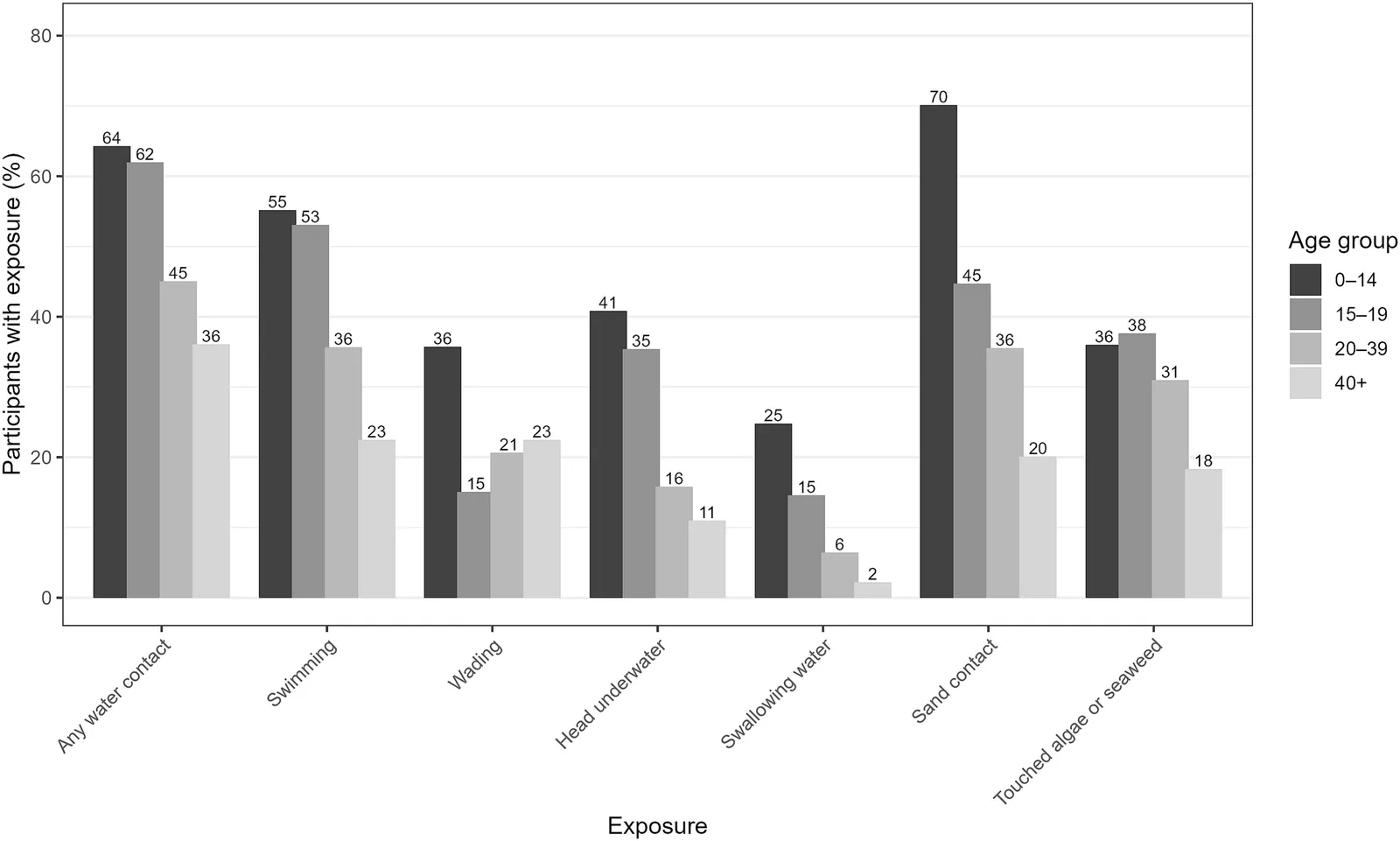
Recreational water users’ water and sand exposure across three provinces of Canada, stratified by age group, 2024-2025

**Figure 2:**
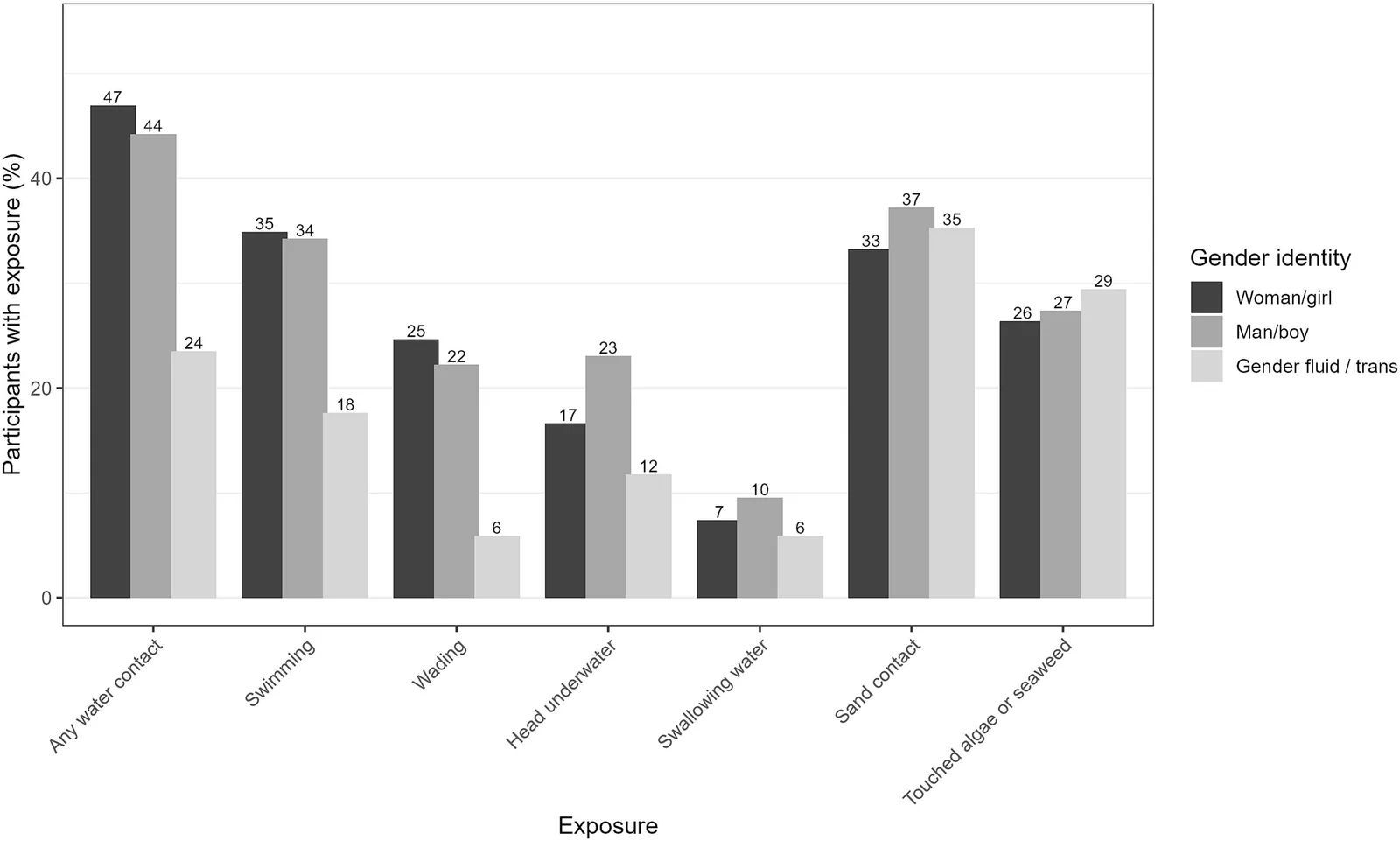
Recreational water users’ water and sand exposure across three provinces of Canada, stratified by gender identity, 2024-2025

**Figure 3:**
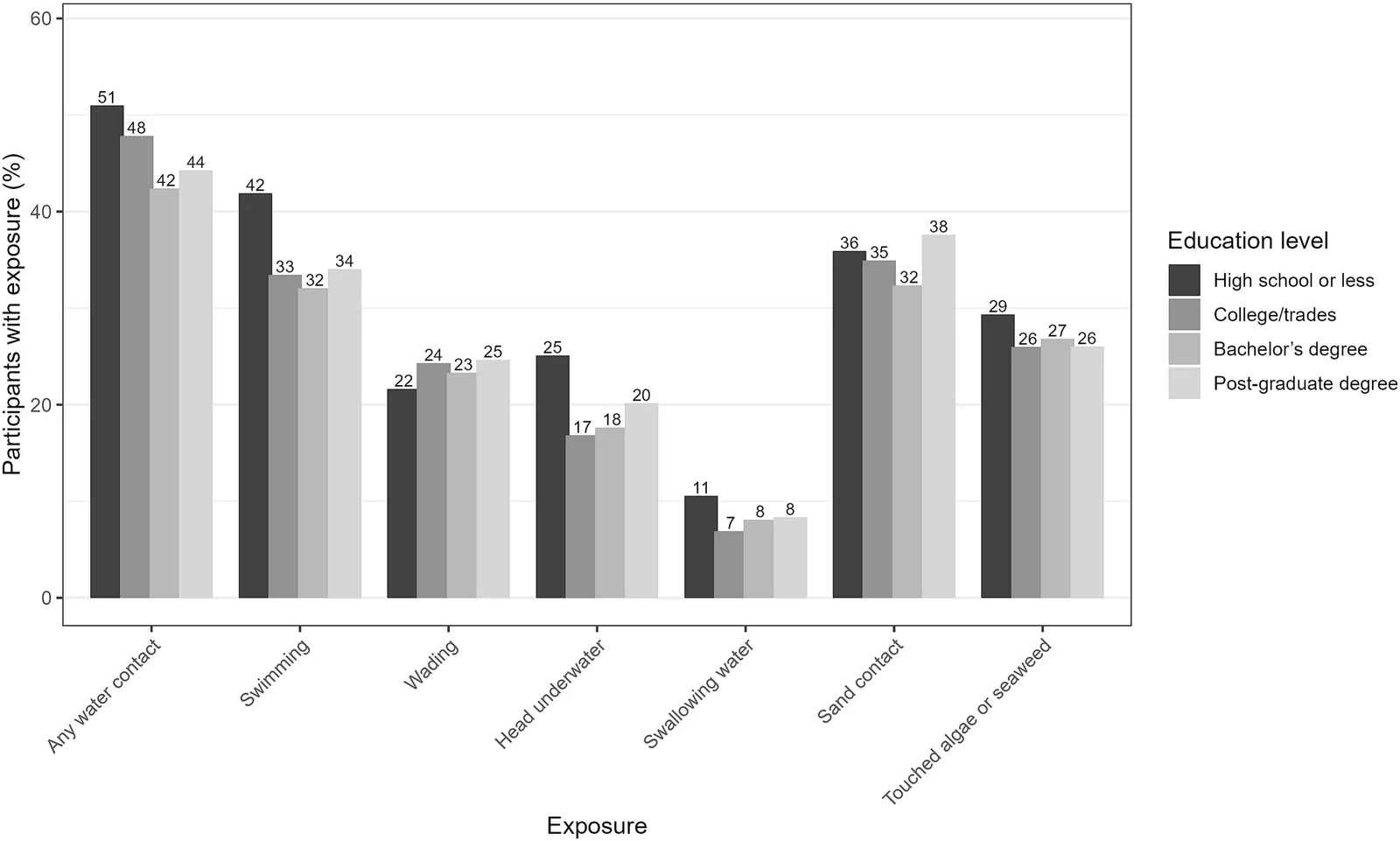
Recreational water users’ water and sand exposure across three provinces of Canada, stratified by higher education completed in household, 2024-2025

**Figure 4:**
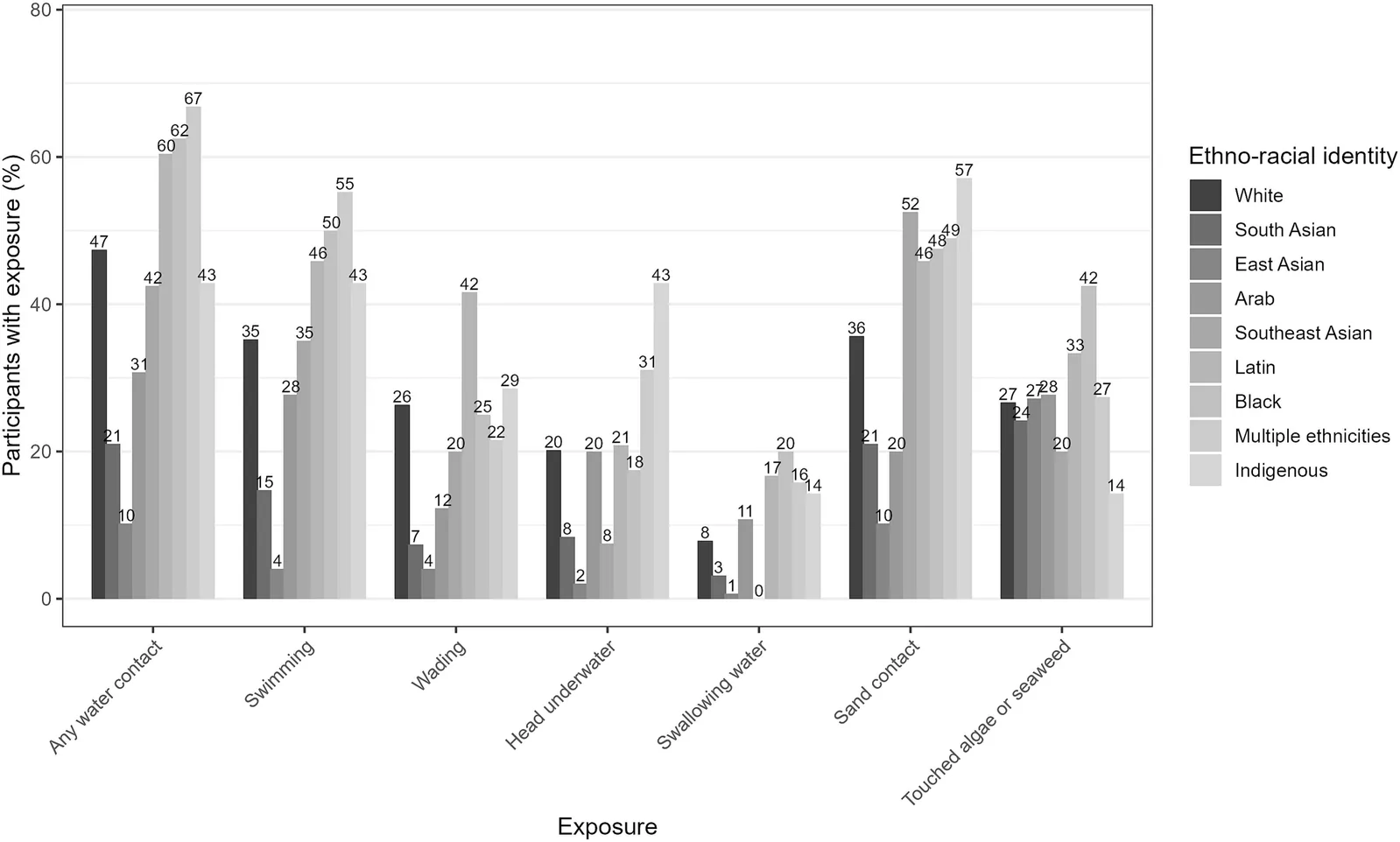
Recreational water users’ water and sand exposure across three provinces of Canada, stratified by Ethno-racial identity, 2024-2025

### 3.3 Human illness outcomes

Illness incidence was low overall, but differences were noted based on water contact exposure (Table 2). AGI occurred in 3.0% of individuals with water contact compared with 1.3% among those without contact. Swallowing water also showed a more pronounced difference: 6.9% vs. 1.6%. Similar exposure-related increases were observed for respiratory symptoms (4.6% vs. 2.0%) and generalized illness (3.4% vs. 2.5%). Skin and eye infections were rare (<2%) (Table 2).

**Table 2:** Incidence of recreational water illness outcomes among recreational water users across three provinces of Canada, stratified by water contact and swallowing water status, 2024-2025

Illness	Water contact N (%)		Swallowing water N (%)		Total N (%) (N = 1470)
	Yes (N = 701)	No (N = 769)	Yes (N = 131)	No (N = 1339)	
AGI	21 (3.0)	10 (1.3)	9 (6.9)	22 (1.6)	31 (2.1)
Respiratory Illness	32 (4.6)	15 (2.0)	12 (9.2)	35 (2.6)	47 (3.2)
Skin Infection	14 (2.0)	8 (1.0)	0 (0.0)	8 (0.6)	22 (1.5)
Eye Infection	4 (0.6)	4 (0.5)	6 (4.6)	16 (1.2)	8 (0.5)
Generalized Illness	24 (3.4)	19 (2.5)	8 (6.1)	35 (2.6)	43 (2.9)

### 3.4 Dogs’ characteristics and exposure

A total of 256 dogs participated in the study, with more than half (56%) coming from the Halifax site (Table 3). The age distribution was consistent across locations, with the majority of dogs falling within the adult category of 3 to 7 years old (48–60%). The sex distribution was well balanced overall, and a significant proportion of the dogs were neutered or spayed (74–88% across the sites). Chronic health conditions were relatively uncommon, reported in only 5–14% of dogs, depending on the site. Most dogs interacted with water or sand in some capacity, although the frequency and type of exposure varied significantly by location. Dogs from Halifax exhibited the highest levels of water contact and ingestion, whereas those from Port Perry experienced much more limited exposure (Table 3). On average, dogs spent 18 minutes in the water, with a range of 1 to 120 minutes. Time spent in water was similar across sexes, though there was considerable variability (see Figure S1). As with humans, dog exposure patterns varied minimally by sex: male dogs were only slightly more likely to enter the water (Figure 5).

**Table 3:** Recreational water user dogs’ characteristics and exposure across three provinces of Canada, stratified by beach, 2024-2025

Characteristic	Manitoba (2024) N (%) (N = 56)	Halifax (2025) N (%) (N = 144)	Port Perry – ON (2024) N (%) (N = 35)	Windsor-Essex-ON (2025) N (%) (N = 21)	Total N (%) (N = 256)
Total participants	56 (21.9)	144 (56.3)	35 (13.7)	21 (8.2)	256 (100)
Age					
<1	4 (7.1)	11 (7.6)	0 (0.0)	0 (0.0)	15 (5.9)
1 – 2	12 (21.4)	34 (23.6)	7 (20.0)	5 (23.8)	58 (22.7)
3 – 7	31 (55.4)	70 (48.6)	21 (60.0)	9 (42.9)	131 (51.2)
>8	9 (16.1)	29 (20.1)	7 (20.0)	7 (33.3)	52 (20.3)
Sex					
Female	28 (50.0)	67 (46.5)	12 (34.3)	9 (42.9)	116 (45.3)
Male	28 (50.0)	77 (53.5)	23 (65.7)	12 (57.1)	140 (54.7)
Neutered or spayed	49 (87.5)	124 (86.1)	26 (74.3)	17 (81.0)	216 (84.4)
Chronic health condition	3 (5.4)	8 (5.6)	3 (8.6)	3 (14.3)	17 (6.6)
Exposure					
Any water contact	36 (64.3)	115 (79.9)	5 (14.3)	12 (57.1)	168 (65.6)
Swallowed water	33 (58.9)	102 (70.8)	2 (5.7)	7 (33.3)	144 (56.3)
Ate debris/seaweed/sand	31 (55.4)	53 (36.8)	7 (20.0)	4 (19.0)	95 (37.1)
Dug in the sand	32 (57.1)	42 (29.2)	4 (11.4)	7 (33.3)	85 (33.2)

**Figure 5:**
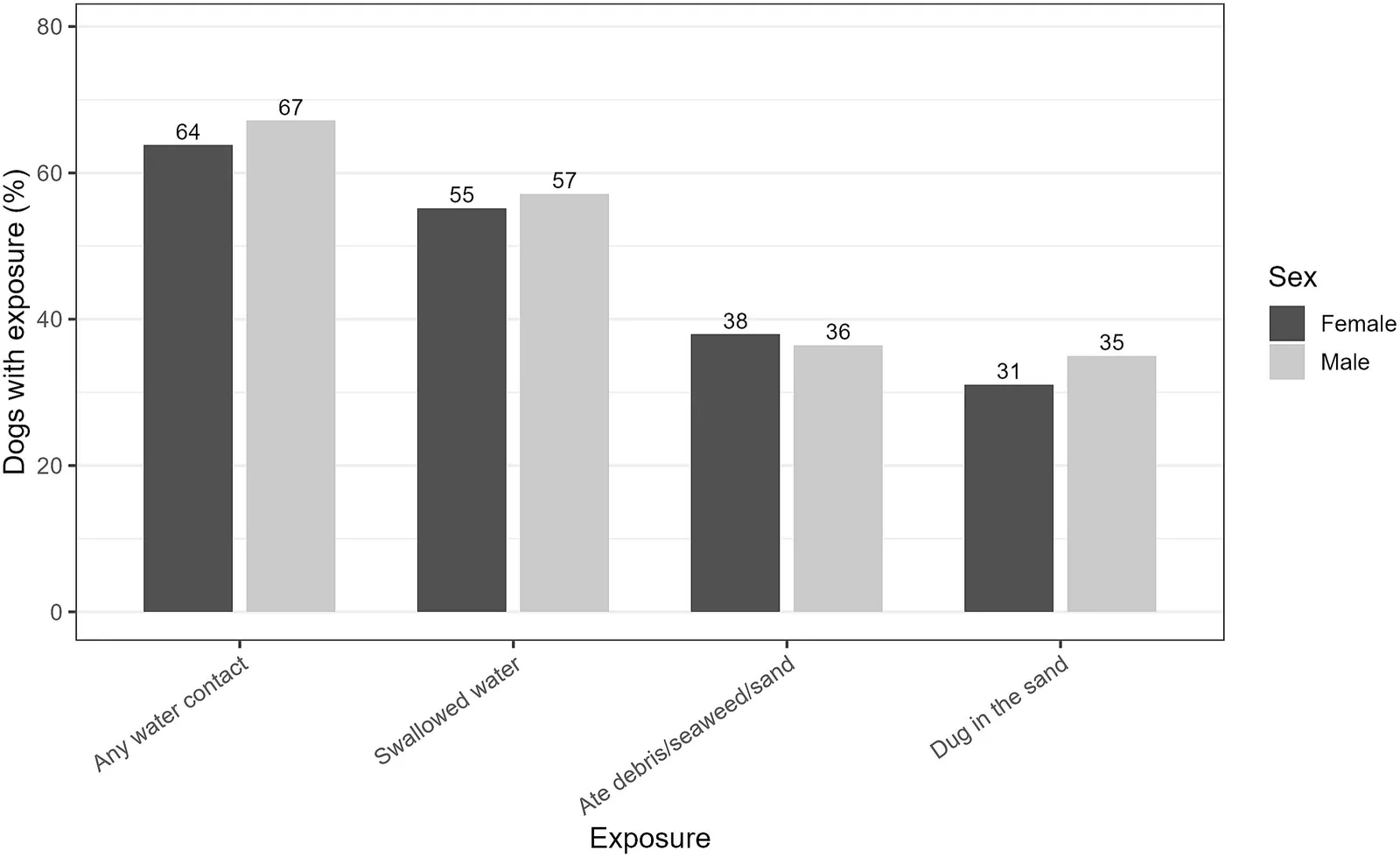
Recreational water user dogs’ water and sand exposure across three provinces of Canada, stratified by sex, 2024-2025

### 3.5 Dog illness outcomes

Overall, the incidence of gastrointestinal, skin, and generalized symptoms was low among dogs. No neurological symptoms were reported (Table 4).

**Table 4:** Incidence of recreational water illness outcomes among recreational water user dogs across three provinces of Canada, stratified by water contact and swallowing status, 2024-2025

Characteristic (N = 160)	Water contact N (%)		Swallowed water N (%)		Total N (%) (N = 160)
	Yes (N = 116)	No (N = 44)	Yes (N = 104)	No (N = 56)	
AGI	3 (2.6)	0 (0.0)	3 (2.9)	0 (0.0)	3 (1.9)
Neurological symptoms	0 (0)	0 (0)	0 (0)	0 (0)	0 (0)
Skin infection/ rash	3 (2.6)	0 (0.0)	3 (2.9)	0 (0.0)	3 (1.9)
Generalized illness	2 (1.7)	0 (0.0)	1 (1.0)	1 (1.8)	2 (1.3)

### 3.6 Cyanobacterial indicators and microbial independence

During the recruitment period, few visible bloom events was observed; however, because bloom identification during screening was based on visual observations, dispersed or non-visible blooms may not have been detected. Only one event was noted in Port Perry, during the late summer. A few events occurred in the Windsor-Essex region, but these were not observed at the beach; rather, they took place in the nearby harbour, where no direct water contact activities occur.

Cyanobacterial concentrations varied widely between the study sites and sampling dates (Figure 6). Exceptionally, only a few samples from Halifax exceeded 1,000 cells/100 mL, while Windsor-Essex saw a significant increase in late summer, with values peaking above 6,000 cells/100 mL on some days. The error bars indicate the differences between replicate sample values from the two sampling locations at each beach across all sites (Figure 6).

**Figure 6:**
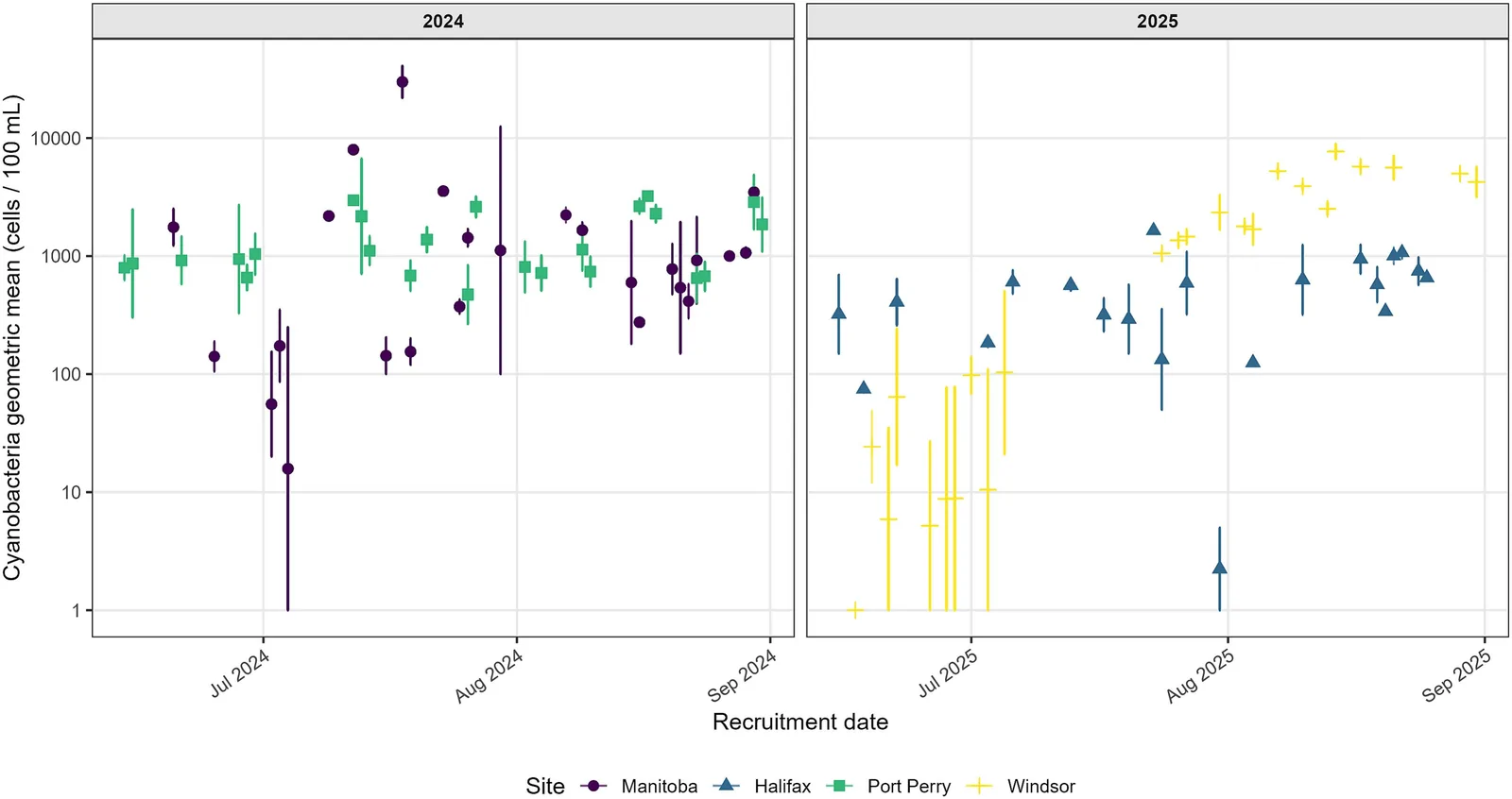
Cyanobacterial geometric mean concentration (cells/100mL) by recruitment date across four sites in Canada, 2024-2025

Test strips yielded negative results on nearly all survey days, with only two positive findings. However, samples were also collected when data collectors observed visible bloom activity and for a few days at the end of the season at each site. Out of the 27 samples sent to the laboratory, approximately 41% showed average microcystin concentrations below the laboratory detection limit of 0.10 μg/L. The remaining samples were also relatively low, with the highest concentration recorded at 0.62 μg/L. This level remains significantly below the established health risk threshold of 10 μg/L [31].

Correlation analyses showed no meaningful association between cyanobacteria and *E. coli* counts (r = –0.02), indicating distinct environmental drivers. Cyanobacteria showed a moderate positive correlation with the phycocyanin–chlorophyll a ratio (r = 0.31), consistent with cyanobacterial dominance during bloom conditions, while *E. coli* counts showed only a weak correlation (r = 0.10).

## 4 Discussion

This study represents one of the first comprehensive assessments across multiple provinces, encompassing both human and dog data on recreational exposure to cyanobacterial risks in Canadian freshwater environments. The primary contribution of this research lies in the characterization of contact behaviours and health outcomes for both humans and companion animals, as well as the environmental conditions and microbial indicators, across various different sites and provinces. Additionally, it explores how these factors intersect within a One Health framework. By jointly investigating recreational patterns, illness trends, and water quality for both humans and dogs, our findings enhance understanding of the interconnected pathways of cyanobacterial risk among humans, animals, and the environment, in line with One Health principles. Notable strengths of this study include its prospective, multi-site design; the integration of behavioural, health, and environmental data; and the employment of both pigment-based and microscopic metrics for cyanobacteria.

Recreational activities among humans varied markedly across sites, reflecting how local geography, beach infrastructure, and cultural patterns of use shape exposure opportunities. Sites characterized by accessible shorelines, sandy substrates, and an established beach-going culture showed substantially higher levels of swimming and immersion, consistent with evidence that beach characteristics, facilities and access, and social norms jointly shape recreational water-contact behaviour [[21], [32], [33]]. In our study, the beaches in Manitoba exhibited the highest levels of swimming and sand contact. In contrast, Halifax beaches saw significantly fewer swimmers, likely due to differences in recreational expectations and the sites' functions, rather than solely to environmental conditions. In Ontario, Port Perry Kinsmen Beach is designated as a non-swimming area and is consistently marked with a blue-green algae warning sign. This includes a permanently posted ‘awareness’ sign, which is replaced by a ‘warning’ sign when a blue-green algae advisory is issued because water sample results exceed Health Canada's Recreational Water Guidelines. Despite this designation, the beach continues to attract various secondary water contact activities, such as boating, kayaking, and fishing. These patterns highlight that recreational exposure does not require full immersion, as contact may also occur through splashing, incidental water ingestion, or contact with the skin and mucous membranes [34]. Consequently, risk communication and preventive strategies should address these varied exposure pathways, particularly for children and individuals participating in water-based sports.

The pronounced heterogeneity observed across various demographic subgroups underscores the influence of social determinants on recreational exposure. Notably, children and teenagers exhibit the highest levels of high-contact behaviours, including immersion, submersion of the head underwater, water ingestion, and sand play. These behaviours are recognized for their propensity to elevate exposure to water-borne hazards and health risks [[35], [36], [37]]. These age-related patterns were evident even at beaches with relatively low cyanobacterial concentrations and minimal bloom occurrence, suggesting that behaviour-driven exposure, rather than bloom magnitude alone, plays a key role in determining contact frequency [[21], [38]]. Sociodemographic gradients were also apparent: individuals from households with lower educational attainment and several ethno-racial groups engaged more frequently in high-contact behaviours, echoing prior research showing that environmental exposures are unevenly distributed across populations [39]. These findings emphasize the need for prevention and risk communication strategies tailored to behavioural, cultural, and socioeconomic contexts: an emerging theme for One Health issues [[40], [41]].

Dogs added an important and often overlooked dimension to this research area. This study found that dogs are frequently exposed to cyanobacteria, although information about illnesses in dogs remains limited. Recent evidence, however, indicates that dogs in Canada and elsewhere have died from disease due to cyanobacteria exposure in water (e.g., [[16], [42]]). It is crucial to minimize dogs' exposure to cyanobacteria. Dog behaviours, such as drinking lake water, digging in sand, and ingesting natural materials, create pathways for microbial and toxin exposure that correspond, but do not perfectly mirror, human exposure [43]. Their inclusion in recreational water guidance may therefore complement a One Health approach that considers human, animal, and ecosystem health. At beaches with high recreational use, satellite-based remote sensing, calibrated and validated for local conditions, could complement routine monitoring by improving the spatial and temporal detection of cyanobacterial blooms and helping target in situ sampling [[44], [45], [46], [47]].

The incidence of AGI was low across sites; however, exposure gradients consistent with ingestion-mediated risk were observed. Participants who engaged in any water contact or who swallowed water reported higher AGI prevalence than those without such exposures. Although ingestion is recognized in the broader literature as an essential pathway for a range of recreational water hazards, including cyanotoxins [[19], [48], [49]], these patterns may reflect cyanobacterial exposure, co-exposures, or other microbial pathways or behavioural factors that influence exposure pathways (e.g., high-contact recreational activities). AGI in dogs, although rare, followed similar directional trends, reinforcing the idea that humans and companion animals share exposure pathways in cyanobacterial bloom-prone freshwater recreational water bodies [[1], [50]].

Our environmental data show substantial spatio-temporal variation in cyanobacterial cell counts. The variability of cyanobacterial peaks emphasizes the importance of longitudinal studies to link exposure with health outcomes and enhance One Health surveillance across human, animal, and environmental data. The observed lack of correlation between cyanobacteria and *E. coli* counts suggests that these indicators reflect distinct recreational water-quality hazards. Under the Canadian recreational water quality guidelines, *E. coli* is used as a primary indicator of fecal contamination in freshwater, with elevated concentrations indicating an increased potential for fecally derived pathogens, including pathogenic bacteria [51]. However, *E. coli* does not reliably predict the presence or absence of individual pathogens and does not necessarily reflect cyanobacterial bloom dynamics, consistent with previous studies documenting the ecological independence of these indicators [[52], [53]]. In Ontario, the moderate positive correlation between cyanobacteria and the phycocyanin:chlorophyll-a ratio further supports the usefulness of pigment-based metrics for tracking bloom activity. Also, the inclusion of a handheld fluorometer to generate pigment-based metrics in this study was intended to evaluate whether a rapid, cost-effective tool could help public health agencies identify early bloom development before a harmful algal bloom becomes visible, an approach increasingly supported in the literature [[29], [54], [55]]. These results support considering fecal contamination and cyanobacterial blooms as distinct recreational water quality concerns. Accordingly, monitoring strategies should reflect the range of hazards relevant to each water body rather than relying only on *E. coli* alone, consistent with Canadian recreational water quality guidelines recommending site-specific monitoring based on known or suspected water quality concerns. As a result, sampling varies across the country and is often limited; for example, Manitoba conducts only monthly proactive sampling, and Ontario and Nova Scotia sample reactively only when a bloom is visually apparent. Expanding proactive monitoring for waterbodies that are at risk, coupled with tools such as remote sensing or satellite-based chlorophyll detection, currently available only for select lakes in Canada, could substantially enhance real-time assessment of cyanobacterial risks [[44], [56], [57]].

This study has several limitations. Behavioural and symptom data were self-reported and subject to recall error biases. Water quality was measured only on recruitment days; thus, possible fluctuations in cyanobacterial abundance or toxin concentrations, noted in bloom dynamics [[58], [59], [60], [61], [62]], may have been missed. In addition, we identified blooms during screening based on visible surface accumulations or water discoloration; therefore, we may not have detected dispersed or non-visible blooms. Dogs were enrolled only from households participating in the human survey, potentially limiting the sample's representativeness, and only one location in Halifax was designated as a dog-specific beach, limiting recruitment opportunities. To address this issue, we focused on recruiting dog owners and families with children. Due to resource limitations, our cyanotoxin sampling was limited to total microcystins, one of many possible cyanotoxins, and this was quantified only occasionally when blooms were suspected. For the purposes of this study, these measurements were descriptive, and we did not assess associations between microcystin concentrations and self-reported health outcomes. Accordingly, the health outcomes documented through the survey should be interpreted as self-reported outcomes following recreational water exposure rather than as effects attributable specifically to microcystins. Other cyanotoxins and cyanobacterial bioactive compounds, including those with neurotoxic or dermatological effects, may also occur during cyanobacterial blooms and could contribute to the range of reported symptoms. Future work should integrate higher-frequency environmental sampling using molecular markers for cyanobacteria and toxins, enhanced symptom follow-up, and expanded veterinary surveillance. Despite these limitations, the study provides a rare, multi-species, multi-province characterization of recreational exposure and cyanobacterial bloom conditions across Canadian beaches.

## 5 Conclusion

Through a One Health perspective, this study underscores that freshwater recreational areas represent environments where human, animal, and microbial systems converge. The findings highlight the need for simple, targeted exposure-mitigation measures for high-contact groups and point to the value of dogs as potential sentinels of environmental risk. Given the variability in recreational behaviour and cyanobacterial conditions across sites, monitoring programs should incorporate multiple indicators rather than relying solely on fecal bacteria. By integrating data from human, animal, and environmental sources, we can achieve a more precise understanding of exposure pathways and associated risks of illness. As climate change and land use continue to exacerbate cyanobacterial bloom activity in Canadian waters, it is crucial to implement coordinated surveillance, improve cross-sector communication, and formulate One Health-aligned risk management strategies to safeguard both human and animal health.

## CRediT authorship contribution statement

**Binyam N. Desta:** Writing – review & editing, Writing – original draft, Visualization, Software, Project administration, Methodology, Investigation, Formal analysis, Data curation, Conceptualization. **Jordan Tustin:** Writing – review & editing, Supervision, Project administration, Methodology, Investigation, Funding acquisition, Conceptualization. **Rachel Jardine:** Writing – review & editing, Supervision, Resources, Project administration, Methodology, Investigation, Conceptualization. **Claire Holeton:** Writing – review & editing, Resources, Methodology, Funding acquisition. **Teresa Brooks:** Writing – review & editing, Funding acquisition. **Elizabeth Montgomery:** Writing – review & editing, Resources, Project administration. **Dylan Lyng:** Writing – review & editing, Resources, Project administration, Funding acquisition. **J. Johanna Sanchez:** Writing – review & editing, Project administration, Methodology, Investigation, Funding acquisition, Conceptualization. **Fatih Sekercioglu:** Writing – review & editing, Methodology, Funding acquisition, Conceptualization. **Ian Young:** Writing – review & editing, Visualization, Supervision, Software, Resources, Project administration, Methodology, Investigation, Funding acquisition, Formal analysis, Data curation, Conceptualization.

## Funding

This work was supported by funding from the 10.13039/100011094Public Health Agency of Canada (principal investigators: IY and JT; grant number: 2324-HQ-000024). The funder had no role in study design, data collection, data analysis, data interpretation, or report writing.

## Declaration of competing interest

The authors declare the following financial interests/personal relationships which may be considered as potential competing interests:

Ian Young reports financial support was provided by Public Health Agency of Canada. If there are other authors, they declare that they have no known competing financial interests or personal relationships that could have appeared to influence the work reported in this paper.

## Data Availability

The deidentified dataset will be made available after the study team completes all planned analyses at the following publicly available GitHub repository: https://github.com/bndesta/algal_bloom
