# Supplementary material for: Cyanobacterial blooms at Canadian freshwater beaches: A prospective cohort study of exposure, health outcomes, and One Health implications
Source: One Health. 2026 Oct 9;23:101608. doi: 10.1016/j.onehlt.2026.101608 (PMC13691274; doi:10.1016/j.onehlt.2026.101608)
Supplement: Supplementary file 1 [file mmc1.docx]

Table S1. Recreational water users’ water and sand exposure across three provinces of Canada, stratified by site, 2024-2025

| Characteristic | Manitoba (2024)  N (%)  (N = 958) | Halifax (2025)  N (%)  (N = 278) | Port Perry – ON (2024)  N (%)  (N = 523) | Windsor-Essex-ON (2025)  N (%)  (N = 565) | Total  N (%)  (N = 2324) |
| --- | --- | --- | --- | --- | --- |
| Any water contact | 661 (69.0) | 42 (15.1) | 43 (8.2) | 316 (55.9) | 1,062 (45.7) |
| Swimming | 526 (54.9) | 24 (8.6) | 22 (4.2) | 232 (41.1) | 804 (34.6) |
| Wading | 338 (35.3) | 29 (10.4) | 4 (0.8) | 171 (30.3) | 542 (23.3) |
| Paddleboarding | 40 (4.2) | 1 (0.4) | 7 (1.3) | 9 (1.6) | 57 (2.5) |
| Boating | 43 (4.5) | 4 (1.4) | 8 (1.5) | 20 (3.5) | 75 (3.2) |
| Kayaking | 34 (3.5) | 3 (1.1) | 5 (1.0) | 8 (1.4) | 50 (2.2) |
| Fishing | 27 (2.8) | 2 (0.7) | 11 (2.1) | 9 (1.6) | 49 (2.1) |
| Diving | 16 (1.7) | 0 (0.0) | 1 (0.2) | 5 (0.9) | 22 (0.9) |
| Canoeing | 9 (0.9) | 4 (1.4) | 7 (1.3) | 2 (0.4) | 22 (0.9) |
| Snorkelling | 13 (1.4) | 0 (0.0) | 1 (0.2) | 1 (0.2) | 15 (0.6) |
| Wakeboarding | 7 (0.7) | 0 (0.0) | 7 (1.3) | 0 (0.0) | 14 (0.6) |
| Waterskiing | 8 (0.8) | 0 (0.0) | 5 (1.0) | 0 (0.0) | 13 (0.6) |
| Other water sports^1^ | 21 (2.1) | 3 (1.1) | 5 (1.0) | 11 (1.9) | 40 (1.6) |
| Head underwater | 323 (33.7) | 7 (2.5) | 11 (2.1) | 114 (20.2) | 455 (19.6) |
| Swallowing water | 115 (12.0) | 11 (4.0) | 7 (1.3) | 60 (10.6) | 193 (8.3) |
| Sand contact^2^ | 475 (49.6) | 50 (18.0) | 22 (4.2) | 267 (47.3) | 814 (35.0) |
| Touched algae or seaweed | 282 (29.4) | 14 (5.0) | 138 (26.4) | 189 (33.5) | 623 (26.8) |

^1^Other water sports included Surfing (N = 7 in Manitoba; N = 3 in Ontario), Sailing (N = 2 in Manitoba; N = 1 in Halifax; N = 5 in Ontario), Kitesurfing (N = 3 in Manitoba; N = 1 in Ontario), Windsurfing (N = 1 in Manitoba).

^2^We defined sand contact as digging in the sand or burying oneself in it.

Figure S1. Recreational water user dogs’ time spent in water across three provinces of Canada, stratified by sex, 2024-2025
